# Relapse of adult B-cell acute lymphoblastic leukemia in the bilateral lacrimal glands: A case report and literature review

**DOI:** 10.1097/MD.0000000000048025

**Published:** 2026-03-13

**Authors:** Hyo Ju Jang, Ga-Young Song, Sung Sun Kim, Kyung-Chul Yoon, Hyeon-Jeong Yoon

**Affiliations:** a Department of Ophthalmology, Chonnam National University Medical School and Hospital, Gwangju, Republic of Korea; b Department of Hematology-Oncology, Chonnam National University and Hwasun Hospital, Hwasun, Jeollanam-do, Republic of Korea; c Department of Pathology, Chonnam National University Medical School and Hospital, Gwangju, Republic of Korea.

**Keywords:** extramedullary relapse, hematological malignancy, lacrimal tumor, orbital tumor

## Abstract

**Rationale:**

Extramedullary relapse of acute lymphoblastic leukemia (ALL) involving the orbit is uncommon, and lacrimal gland infiltration is particularly rare in adults. Bilateral lacrimal gland enlargement can be clinically nonspecific and may be misinterpreted as inflammatory or autoimmune disease, potentially delaying diagnosis. This case report describes a rare adult case of extramedullary relapse of B-cell ALL presenting as bilateral lacrimal gland enlargement following allogeneic hematopoietic stem cell transplantation (HSCT).

**Patient concerns:**

A 55-year-old woman presented with a 4-day history of a painless, palpable mass in the right upper eyelid. The patient had a history of Philadelphia chromosome-negative B-cell ALL and had previously undergone chemotherapy followed by allogeneic HSCT.

**Diagnoses:**

Orbital computed tomography demonstrated diffuse, heterogeneous enlargement of both lacrimal glands, more prominent on the right side. Incisional biopsy of the right lacrimal gland with immunohistochemical staining (terminal deoxynucleotidyl transferase positive and B-lineage markers positive) confirmed extramedullary relapse of B-cell ALL.

**Interventions:**

The patient received salvage chemotherapy followed by a 2nd allogeneic HSCT.

**Outcomes:**

Follow-up orbital computed tomography performed 6 months after treatment showed complete resolution of bilateral lacrimal gland enlargement without evidence of local progression.

**Lessons:**

Bilateral lacrimal gland enlargement in patients with a history of ALL may represent isolated extramedullary relapse, even in the absence of hematologic abnormalities. Early histopathologic confirmation is essential to prevent diagnostic delay and to enable timely systemic management in ALL patients.

## 1. Introduction

Leukemia is a hematological malignancy characterized by the clonal proliferation of white blood cells in the bone marrow (BM), peripheral blood, and various organs. It is classified as acute lymphoblastic leukemia (ALL), acute myeloid leukemia, chronic lymphocytic leukemia, and chronic myeloid leukemia, based on the origin of the affected cells and disease progression.^[1,2]^ Among these, ALL is composed of immature lymphoid cells, predominantly occurs in children, and is less common in adults.^[1,3]^

Although ALL relapse primarily occurs in the BM, it can also involve extramedullary sites, such as the central nervous system or testes. Orbital infiltration, however, is a rare manifestation and has been reported only sporadically.^[4]^ Lacrimal gland involvement in ALL is particularly rare in adults, with most reported cases occurring unilaterally in pediatric patients. In adults, bilateral involvement is virtually nonexistent.

Here, we report a rare case of extramedullary relapse in an adult patient with ALL who presented with bilateral lacrimal gland enlargement. Despite nonspecific clinical and radiological findings, early diagnosis and treatment were achieved through prompt surgical biopsies. Bilateral lacrimal gland enlargement can be easily misinterpreted as an autoimmune or inflammatory condition, such as Sjögren syndrome or granulomatous diseases, or a secondary change due to leukemia, potentially leading to delayed diagnosis, even in patients with a known history of leukemia. This study also includes a systematic review of previously reported cases and literature regarding orbital or lacrimal gland involvement in ALL, and discusses diagnostic approaches for atypical relapses.

## 2. Case presentation

### 2.1. Patient information

A 55-year-old woman presented with a 4-day history of a palpable mass on the right upper eyelid. She was previously diagnosed with Philadelphia chromosome-negative B-cell ALL, 8 months prior to presentation and had been treated with chemotherapy followed by allogeneic hematopoietic stem cell transplantation. At the time of initial diagnosis, she was referred to the hematology department because of thrombocytopenia. Complete blood count revealed a white blood cell count of 5500/µL, hemoglobin level of 11.8 g/dL, and platelet count of 33,000/µL. Peripheral blood smear showed pancytopenia with 30% blasts, and BM examination revealed 88% blasts. The immunohistochemical results were positive for cluster of differentiation (CD)10, CD19, CD34, and human leukocyte antigen–DR.

The patient initially achieved complete remission after chemotherapy (cyclophosphamide, vincristine, doxorubicin, methotrexate, cytarabine, and dexamethasone) and bispecific antibody therapy (blinatumomab). However, a relapse occurred in the nasal cavity, for which additional treatment with an antibody-drug conjugate (inotuzumab) was administered, resulting in a 2nd remission, followed by the 1st allogeneic hematopoietic stem cell transplantation. On day 73 posttransplantation, without evidence of graft-versus-host disease or relapse, the patient presented with a mass on the right upper eyelid. At the 1st ophthalmologic visit, complete blood count revealed leukopenia (white blood cell count, 1700/µL), mild anemia (hemoglobin, 10.6 g/dL), and thrombocytopenia (platelet count, 88,000/µL).

### 2.2. Clinical findings

**Figure 1.**
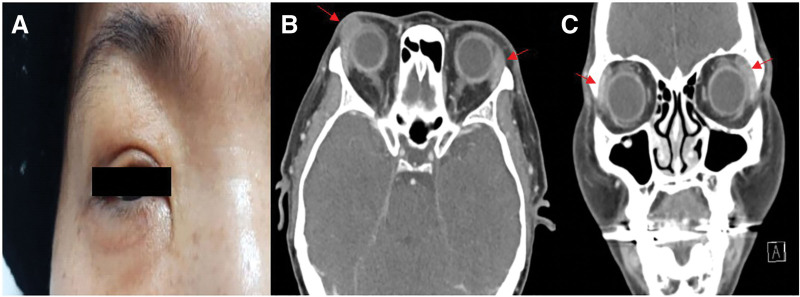
Clinical and radiologic findings at presentation. (A) External photograph of the right upper eyelid showing a painless mass without erythema. (B) Axial and (C) coronal CT images show diffuse, heterogeneous enlargement of both lacrimal glands, more prominent on the right. CT = computed tomography.

Upon examination, her best-corrected visual acuity was 0.8 in the right eye and 0.9 in the left eye. The fundoscopic examination results were unremarkable. A nontender mass was palpable on the right upper eyelid with nonerythematous swelling (Fig. 1A). Computed tomography (CT) showed diffuse bilateral enlargement and heterogeneity of the lacrimal glands (Fig. 1B and C).

### 2.3. Diagnostic and therapeutic interventions

**Figure 2.**
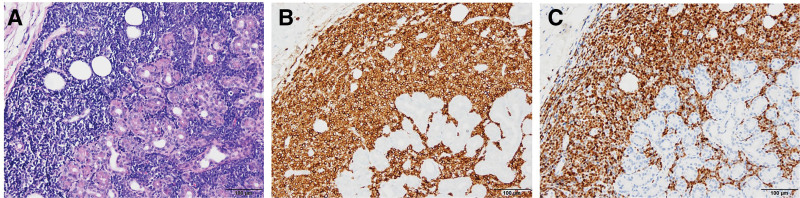
Histopathologic findings of lacrimal gland. (A) Diffuse lymphoblastic cell infiltration in the stroma (hematoxylin and eosin stained, X200). (B and C) The tumor cells are immunopositive for CD79a (X200) and terminal deoxynucleotidyl transferase (TdT) (X200). CD79a = cluster of differentiation 79a.

**Figure 3.**
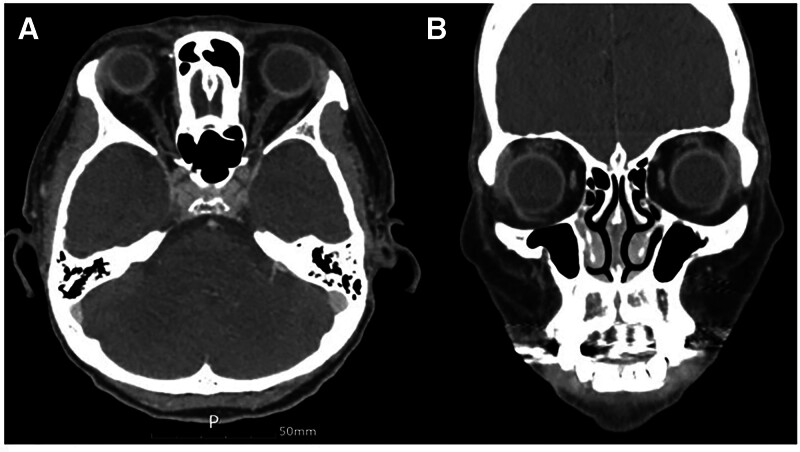
Postchemotherapy CT of the orbit. The axial (A) and coronal views (B) show a decrease in the size of the diffusely enlarged lacrimal glands. CT = computed tomography.

Excisional biopsy of the right upper eyelid mass was performed. Immunohistochemistry revealed CD20 (−), CD3 (−), terminal deoxynucleotidyl transferase (+), Ki-67 (90%–100%), CD10 (+), and CD79a (+), confirming an extramedullary relapse of B-cell ALL (Fig. 2). Subsequent positron emission tomography revealed additional extramedullary lesions in the left submandibular gland and right breast. The patient underwent salvage chemotherapy with mitoxantrone and cytarabine, followed by a 2nd allogeneic hematopoietic stem cell transplant. Six-month follow-up CT showed resolution of the lacrimal gland enlargement (Fig. 3).

### 2.4. Methods of literature review

We conducted a narrative literature review using PubMed and Google Scholar to identify reports of orbital and lacrimal gland involvement in ALL. The search terms included various combinations of “ALL,” “orbit,” “ocular,” “lacrimal gland,” “lacrimal sac,” “orbital mass,” and “extramedullary relapse.” The search was limited to literature published up to June 2025. We included case reports, case series, and review articles describing leukemic relapse involving the orbit, lacrimal gland, or ocular adnexa. Studies focusing on secondary orbital changes related to hematologic abnormalities, treatment-related complications, or nonleukemic inflammatory conditions were excluded. Duplicate publications, articles with inaccessible full texts, and studies lacking sufficient clinical detail were also excluded. Ultimately, 5 review articles and 11 relevant case reports were selected for analysis (Tables 1 and 2) and compared with the present case.^[1–16]^

**Table 1:** Summary of review articles on orbital or lacrimal involvement in leukemia and lacrimal gland tumors.

Author (year)	Review focus	Data type	Key findings	Clinical implications
Chen et al.^[5]^ (2023)	Ocular involvement in adult leukemia	Case series with literature review	Adult leukemic involvement in orbit is rare; most cases respond to systemic chemotherapy.	Orbital infiltration in ALL is rare but awareness is critical.
Hafeez et al.^[6]^ (2019)	Ocular manifestations of acute leukemia	Cross-sectional study	Ocular signs (esp. retinal hemorrhages) observed in 48% of patients; more frequent in AML than ALL	Routine ophthalmic screening is recommended in acute leukemia.
Mombaerts et al.^[7]^ (2024)	Metastatic tumors to the lacrimal gland	Retrospective study and literature review	Describes clinical and imaging features of metastatic lacrimal gland lesions.	Biopsy is necessary to differentiate metastatic lesions, even when they appear benign.
Aryasit et al.^[8]^ (2023)	Clinicopathologic characteristics of lacrimal gland tumors	Retrospective cases series	Comprehensive review of histologic and imaging features of various lacrimal gland tumors.	Biopsy is essential for diagnosis of lacrimal gland masses.
Rose^[9]^ (2009)	Diagnostic strategies for lacrimal gland tumors	Expert opinion	Biopsy may disrupt tumor capsule and increase recurrence risk in suspected malignancies.	Consider complete excision rather than biopsy when malignancy cannot be excluded.

ALL = acute lymphoblastic leukemia, AML = acute myeloblastic leukemia.

**Table 2:** Summary of published cases of ALL with orbital or lacrimal involvement.

Author (year)	Age	Site of involvement	Laterality	Diagnosis	BM involvement	Disease phase	Notes
Alsalem et al.^[1]^ (2021)	Pediatric	Orbit	Unilateral	Biopsy	Yes	Initial	
Gounder et al.^[2]^ (2022)	Pediatric	Orbit	Unilateral	Biopsy	Yes	Initial	
Stevanovic and Yoon^[3]^ (2023)	Adult	Lacrimal gland	unilateral	Biopsy	No	Relapse	
Johnston^[4]^ (2003)	Pediatric	Lacrimal gland	Bilateral	Biopsy	Yes	Relapse	
Sathitsamitphong et al.^[10]^ (2019)	Pediatric	Orbit	Unilateral	Biopsy	Yes	Initial	
Huh et al.^[11]^(2002)	Adult	Orbit	Unilateral	Biopsy	Yes	Relapse	
Levitt et al.^[12]^ (2021)	Pediatric	Lacrimal sac	Bilateral	Biopsy	Yes	Relapse	
Zhou and Behdad^[13]^ (2017)	Adult	Orbit	Unilateral	Biopsy	No	Relapse	Mixed lineage ALL with orbital mass
Esmaeli et al.^[14]^ (2001)	Adult	Orbit	Unilateral	Biopsy	Yes	Relapse	T-ALL relapse with orbital mass
Dini et al.^[15]^ (2023)	Adolescent	Optic nerve	Unilateral	Imaging only	Yes	Relapse	
Cardone et al.^[16]^ (2006)	Adolescent	Lacrimal gland	Unilateral	Biopsy	No	Relapse	

ALL = acute lymphoblastic leukemia, BM = bone marrow, T-ALL = T-cell acute lymphoblastic leukemia.

## 3. Discussion

Orbital lesions have various causes including infections, inflammation, or neoplasms. Leukemic or lymphomatous infiltration is a rare but important differential diagnosis. A literature review by Teresa et al reported 29 cases of orbital infiltration by acute myeloid leukemia and only 3 cases by ALL.^[5]^ Similarly, an analysis of 1264 orbital lesions at Wills Eye Hospital identified 130 cases related to lymphoma or leukemia, of which only 2 cases were confirmed to be ALL, highlighting its rarity.^[17]^

Orbital infiltration by ALL is often sporadic and atypical. In addition to direct leukemic infiltration, secondary changes due to hematologic abnormalities, immunosuppression, or chemotherapy-related complications may also manifest.^[1,6]^ Hafeez et al reported that such secondary changes are more common than direct infiltration and that lesions often improve following systemic recovery.^[6]^ However, in some cases, orbital involvement may represent an early sign of relapse, highlighting the importance of histopathological confirmation.

Lacrimal gland enlargement, as seen in this case, may result from inflammation, infection, or neoplasia and may have an asymptomatic or nonspecific presentation. Imaging of leukemic orbital infiltration typically shows homogenous soft-tissue masses with a density similar to that of muscle or gray matter, with minimal contrast enhancement on CT or magnetic resonance imaging.^[1,7]^ Therefore, differentiation based only on clinical and radiologic findings is difficult. Surgical biopsy and histopathological examination are essential to confirm relapse and plan treatment.

As summarized in Table 1, previous review articles have emphasized the clinical significance of ophthalmic symptoms and the need for tissue biopsy in patients with a history of leukemia. While some experts caution against performing biopsies for epithelial malignancies of the lacrimal gland owing to the risk of capsular rupture and local recurrence,^[9]^ prompt systemic treatment guided by an accurate histopathological diagnosis is a priority in cases of hematologic malignancy. Even in patients with a poor general condition, biopsy should be strongly considered. There is no standard treatment for orbital infiltration by leukemia. However, immediate chemotherapy is typically initiated, with radiotherapy or stem cell transplantation considered based on the patient condition.^[5,7]^

Lacrimal gland infiltration is particularly rare in patients with orbital infiltration. The incidence of lacrimal gland tumors is approximately 1 case per million people annually, and most are benign, with pleomorphic adenoma being the most common.^[8,9]^ Recent reports describe only a limited number of ALL relapse cases, presenting in various forms, such as an orbital mass, extraocular muscle enlargement, or lacrimal sac involvement. Table 2 summarizes the reported cases of orbital or lacrimal gland infiltration by ALL confirmed by imaging and histopathology. Most cases occurred unilaterally in pediatric patients, whereas relapse with bilateral lacrimal gland enlargement in adults, as seen in this case, was extremely rare.^[1–5,10–17]^

In conclusion, bilateral lacrimal gland enlargement can mimic inflammatory or autoimmune conditions such as dacryoadenitis, which may delay diagnosis. However, in patients with a history of leukemia, new lacrimal gland swelling may represent isolated extramedullary relapse rather than a benign process. Because ophthalmic manifestations can be the earliest or even sole indicator of relapse, prompt histopathological confirmation and systemic evaluation are essential for accurate diagnosis and timely treatment. This case highlights the importance of considering leukemic relapse in patients presenting with atypical orbital findings and emphasizes the need for vigilance in posttransplant ALL follow-up.

## Acknowledgments

Supported by the Chonnam National University Hospital Biomedical Research Institute (BCRI24084). Basic Science Research Program through the National Research Foundation of Korea (NRF) funded by the Ministry of Education (RS-2023-35800280734).

## Author contributions

**Conceptualization:** Kyung-Chul Yoon, Hyeon-Jeong Yoon.

**Data curation:** Hyo Ju Jang, Hyeon-Jeong Yoon.

**Investigation:** Hyo Ju Jang, Sung Sun Kim, Hyeon-Jeong Yoon, Ga-Young Song.

**Methodology:** Hyo Ju Jang, Kyung-Chul Yoon, Hyeon-Jeong Yoon.

**Resources:** Sung Sun Kim.

**Validation:** Sung Sun Kim, Ga-Young Song.

**Visualization:** Hyo Ju Jang, Hyeon-Jeong Yoon.

**Writing – original draft:** Hyo Ju Jang, Hyeon-Jeong Yoon.

**Writing – review & editing:** Hyo Ju Jang, Ga-Young Song, Kyung-Chul Yoon, Hyeon-Jeong Yoon.
